# New global target on non-communicable diseases: a call to action for the global cardiovascular disease community

**Published:** 2012-06

**Authors:** Johanna Ralston

**Affiliations:** Chief Executive Officer, World Heart Federation, Geneva, Switzerland

For many years, cardiovascular disease (CVD) was regarded as a lifestyle disease primarily affecting ageing, affluent populations. This is reflected in the virtual absence of global policies concerned with CVD control in poor and rich countries alike. However, CVD and other non-communicable diseases (NCDs), including cancer, chronic respiratory disease and diabetes, account for nearly two-thirds (63%) of global deaths, with the majority of deaths from NCDs (80%) occurring in low- and middle-income countries (LMICs).1

This represents a public health emergency requiring an urgent worldwide response. Now with the World Health Organisation’s (WHO) adoption of a global target to prevent premature NCD mortality, the time has finally arrived for the global CVD community to join forces in reducing CVD suffering and death in all countries and among all populations.

The World Heart Federation and its members and colleagues in the CVD community have been fighting the burden of diseases for years, while also advocating for governments to act. In the run up to the first-ever United Nations High-Level meeting on NCDs, which took place on 19 September 2011, the World Heart Federation worked with its 200 member organisations globally to lobby for CVD and the other NCDs to be recognised as a priority on the global health and development agendas.

World leaders finally heeded our calls to action and they unanimously adopted a political declaration2 agreeing to address the prevention and control of NCDs worldwide, with an emphasis on developing countries. The declaration highlights NCDs as a major challenge for development in the 21st century, emphasising that NCDs undermine social and economic development, and threaten the achievement of global development and poverty-eradication goals.

Just eight months later, governments agreed to take responsibility for responding to the challenge of NCDs. At the 65th World Health Assembly, all 194 member states agreed to adopt the first ever global NCD target: a 25% reduction in premature mortality from NCDs by 2025.3 The adoption of this bold global target is the result of commitment, hard work and a major lobbying effort from CVD activists. Most important to note, the overall mortality target and additional targets to be adopted later this year place CVD prevention and control at the heart of the NCD agenda, with risk management, low-cost treatment and care of CVD central to achieving the mortality and risk-factor targets.

Many members of the African CVD community are to be congratulated for their efforts to make these achievements possible. In particular, Bongani Mayosi, chair of the World Heart Federation Rheumatic Heart Disease Working Group and, professor and head of the Department of Medicine at the University of Cape Town, participated in a civil society interactive hearing on NCDs at the United Nations in June 2011, to inform preparations for the UN meeting in September. At that High-Level meeting, Dr Kingsley Akinroye, then-president of the African Heart Network and board member of the World Heart Federation, spoke from the floor of the UN General Assembly about the importance of strengthening national policies and capacity to address the control of NCDs.

But we cannot rest now. The target represents a rallying cry for further action by the CVD community. It automatically elevates CVD on the global health policy agenda, providing an ‘opportunity springboard’ from which we can accelerate action to reduce the global CVD burden. Because CVD is responsible for a higher proportion of NCD deaths than cancer, chronic respiratory diseases and diabetes combined (48 vs 36.5%),1 world leaders will look to us and our efforts to help reduce the CVD burden – and so our challenge is great.

The next 13 years are crucial, and in order to mount a comprehensive response for achieving the target, the CVD community must forge innovative partnerships with policy makers, the private sector and healthcare professionals to create strategies that prevent CVD at local, regional and national levels. We need to look beyond the health sector and consider the many factors that influence heart health (including healthy eating, physical activity and tobacco consumption). We need to serve as the catalyst for renewed, global efforts to encourage heart-healthy behaviours. A concerted, global response is vital – we won’t curtail this global epidemic by continuing the same fragmented responses we have followed in the past. Everyone has a crucial role to play, and we must lead the charge and propel them to act.

To achieve the target, policy makers urgently need to take action to help modify behavioural risk factors. Many CVD prevention strategies exist, however governments must do more to ensure that these are fully implemented and well-articulated in NCD plans, which the UN political declaration requires governments to complete by the end of 2013. As an example, one of the success stories in the fight against CVD is the WHO’s Framework Convention on Tobacco Control (FCTC), a treaty addressing issues around tobacco consumption, including restricting sales and advertising.

National smoke-free legislation has been passed in Ghana, Mauritius, Niger, Kenya, Zambia and South Africa.4 However, according to the latest (2010) global progress report on implementation of the FCTC, only 50% of parties in the African region reported implementing a comprehensive ban on tobacco advertising, promotion and sponsorship.5 Our job therefore is to advocate for the full implementation of the FCTC, and at the same time push for more public-health campaigns to educate people on the links between tobacco consumption, CVD and premature death.

Collaboration with the private sector is also imperative. Let us consider access to healthy food as an example. The factors that influence an individual’s ability to eat healthily are many, and are often beyond the control of that individual. In Africa, where many countries are increasingly experiencing a dual burden of obesity and malnutrition, strategies are required not just to affect an individual’s consumption of food but to modify food production processes.

We can spearhead strategies to reformulate food products, to distribute healthy food options to those communities most in need, to promote and foster incentives for fruit and vegetable consumption, and to educate consumers to drive healthy food choices among those who have options available to them. We may face resistance from some corporate leaders, policy makers and even donors, so advocacy to stress the need for action is required alongside the development of new and creative partnerships that meet both health and corporate objectives wherever possible. We must also campaign for companion strategies for policies to change food production in the long term.

We must work with healthcare providers to implement strategies that improve access to care and treatment. Consider blood pressure: in high-income countries, widespread screening, diagnosis and treatment have led to an impressive decrease in mean blood pressure,1,6 and correspondingly a drop in mortality from stroke and coronary heart disease is to be expected. Yet in Africa, more than one in three people (36.8%) are estimated to have raised blood pressure, and the prevalence is increasing.1,6

Improving the availability of screening in primary care and treatment of high blood pressure with affordable essential drugs, including aspirin and statins, will prove vital to reducing premature CVD mortality in developing countries. More research is also needed to understand the burden of hypertension in the region in order to tailor approaches to addressing it. The distribution of penicillin to prevent rheumatic heart disease is a similar strategy that would cost little but have great impact. It is essential that we tackle these global inequalities in order to meet the target.

Although we applaud the progress made at the World Health Assembly, the above examples show the complexity of CVD management. The global target is a landmark achievement that obliges action to deliver change for people with or at risk of NCDs and especially CVD. However, in isolation it is not enough – further targets are needed to shape a more complete framework and better guide collaborative, global action against CVD and its risk factors.

We must keep the pressure on our governments to ensure the best possible outcomes for the millions of people suffering from CVDs and to avoid the 17.3 million deaths that occur each year. Specific targets are being considered for adoption around reducing the consumption of tobacco, salt/sodium, trans fats, and harmful levels of alcohol; reducing physical inactivity, elevated blood pressure, cholesterol and obesity; and ensuring access to affordable, quality-assured essential medicines, including multidrug therapy for people who have been identified at high risk of CVD.

The window of opportunity to change the face of CVD forever is now and throughout the year, since the final targets will be agreed by member states in October 2012. We call on the CVD community to champion the additional targets, and push world leaders to agree on these promptly. Together we can avert deaths from CVD using proven interventions, and save lives around the world.
